# Supplementary material for: Robust genome editing activity and the applications of enhanced miniature CRISPR-Cas12f1
Source: Nat Commun. 2025 Jan 15;16:677. doi: 10.1038/s41467-025-56048-w (PMC11733285; doi:10.1038/s41467-025-56048-w)
Supplement: Supplementary file 1 — Supplementary Information [file 41467_2025_56048_MOESM1_ESM.pdf]

Supplementary Information

Robust genome editing activity and the applications of enhanced miniature CRISPR-Cas12f1

Soo-Ji Park, Sungjin Ju, Won Jun Jung, Tae Yeong Jeong, Da Eun Yoon, Jang Hyeon Lee, Jiyun Yang, Hojin Lee, Jungmin Choi, Hyeon Soo Kim, and Kyoungmi Kim

Supplementary Figures

Supplementary Figure 1. Lipid-based plasmid transfection and indel efficiencies of Cas12f1_v1.

Supplementary Figure 2. Indel frequencies of Cas12f1_v1 variants with modified sgRNA stems and free energy of sgRNA secondary structure.

Supplementary Figure 3. Comparison of gene editing efficiency of Cas12f1 variants with mutated amino acids that interact with the 6th nucleotide of the sgRNA spacer or the 15th nucleotide of the target DNA strand.

Supplementary Figure 4. Indel frequencies of Cas12f1_v3 + G181R and Cas12f1_v3 + G181R + sgRNA_S1b variants.

Supplementary Figure 5. Protein sequence alignment of Un1Cas12f1, AsCas12f, OsCas12f1, and RhCas12f1.

Supplementary Figure 6. Indel efficiency of Cas12f1_v7 variants with mutations introduced to enOsCas12f1 and enRhCas12f1.

Supplementary Figure 7. Comparison of intracellular delivery and gene editing efficiency of SpCas9, AsCpf1, Cas12f1_ge4.1, and eCas12f1 using three different DNA delivery methods.

Supplementary Figure 8. Evaluation of eCas12f1 as a potential breast cancer therapy.

Supplementary Figure 9. Genotypes of *BRAF* gene in A375 and HEK293T cells.

Supplementary Figure 10. Base editing window of eCas12f1-ABE

Supplementary Figure 11. Base editing window of eCas12f1-CBE

Supplementary Tables

Supplementary Table 1. sgRNA spacer sequences for evaluating Cas12f1 variants.

Supplementary Table 2. Target sequences used for comparison between SpCas9, AsCpf1, CasMINI-V3.1, Cas12f1_ge4.1, and eCas12f1.

Supplementary Table 3. Target sequences used for regulation of gene expression.

Supplementary Table 4. Primer sequences for real-time qPCR.

Supplementary Table 5. DNA sequences of eCas12f1.

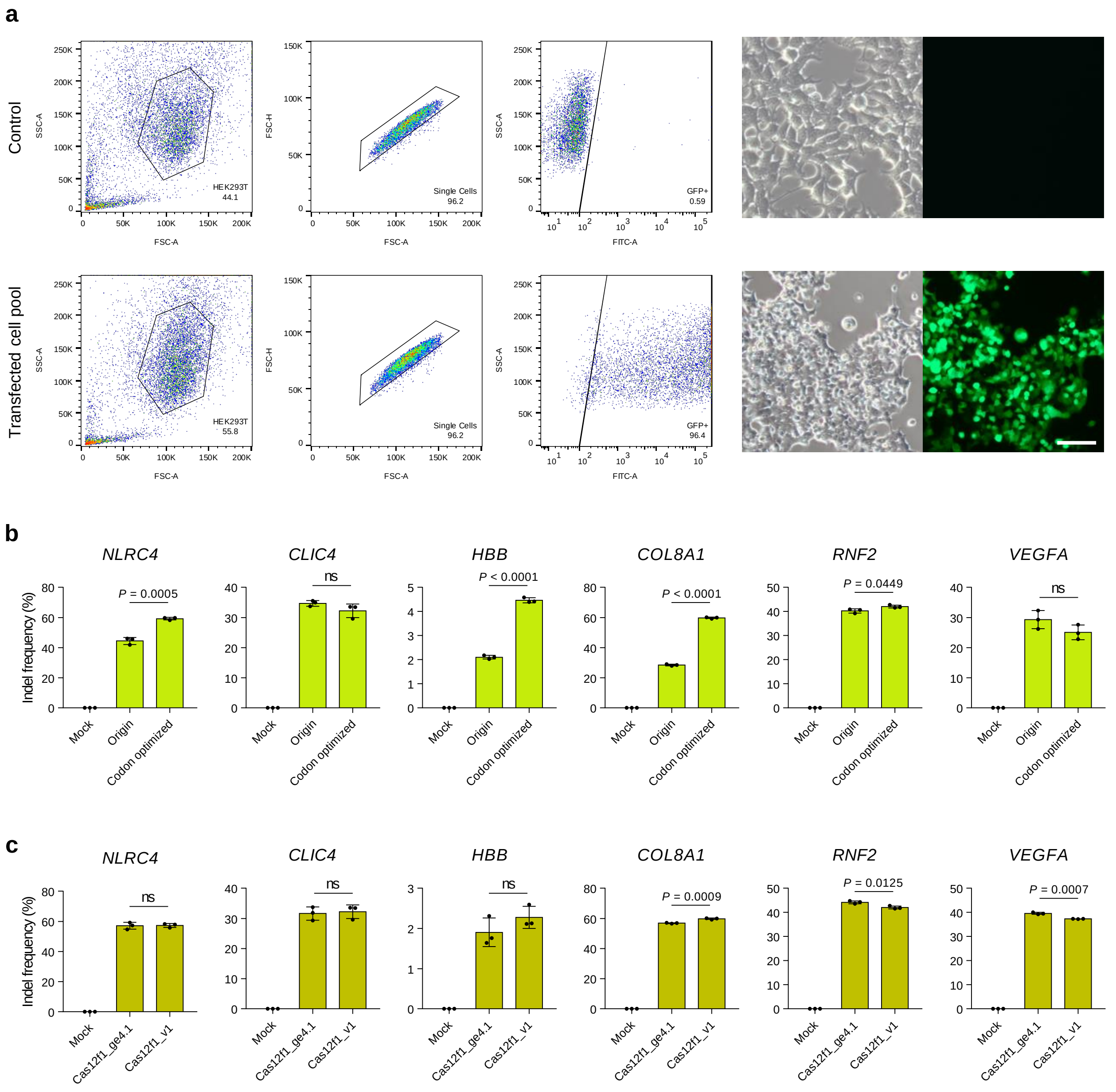

Supplementary Fig. 1 Lipid-based plasmid transfection and indel efficiencies of Cas12f1_v1.

a Analysis of transfection efficiency. Plasmid encoding Cas12f1 and EGFP reporter was transfected into HEK293T cells, and the percentage of GFP-positive cells was analyzed after 72 hr post-transfection through flow cytometry (left panel). GFP-positive cells were also confirmed in fluorescence images (right panel). Scale bar: 50 µm. **b** Indel frequency of Cas12f1 with original or codon optimized sequences of Cas12f1. Gene editing efficiency was confirmed at six targets. **c** Comparison of Indel efficiency between Cas12f1_ge4.1 and Cas12f1_v1 at six targets. Data represent the mean ± s.d. of three independent biological replicates. *P*-values were obtained using the two-tailed Student's t-test. ns, no significant.

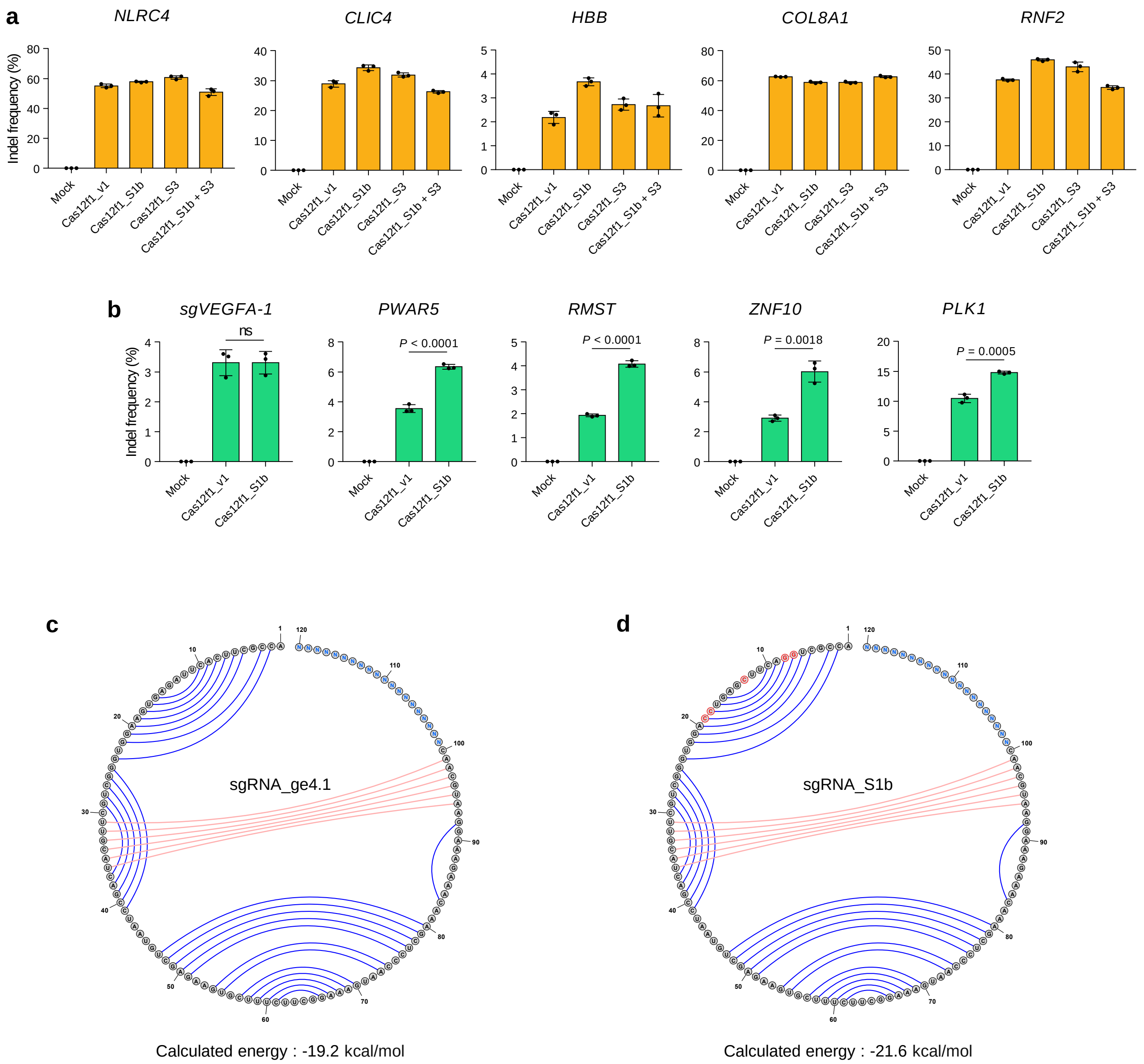

Supplementary Fig. 2 Indel frequencies of Cas12f1_v1 variants with modified sgRNA stems and free energy of sgRNA secondary structure.

a Indel frequency of Cas12f1_S1b, Cas12f1_S3 and the combination of the two variants at five gene targets. **b**, Further investigation of Cas12f1_S1b at five gene targets. **c**,**d** Secondary structures of sgRNA_ge4.1 and sgRNA_S1b drawn using the RNAstructure software package (https://rna.urmc.rochester.edu/RNAstructure.html). RNA sequences are arranged in the circle. Blue lines, base pairs of RNA that form a stem-loop structure; red lines, base pairs formed in the sgRNA secondary structure; red letters, modified nucleotides; blue letters, sgRNA spacer. The free energy of sgRNA_ge4.1 (c) and sgRNA_S1b (d) secondary structures calculated by RNAeval webserver (http://rna.tbi.univie.ac.at/cgi-bin/RNAWebSuite/RNAeval.cgi). Data in the bar graphs represent mean ± s.d. of three independent biological replicates. *P*-values were obtained using the two-tailed Student's t-test. ns, not significant.

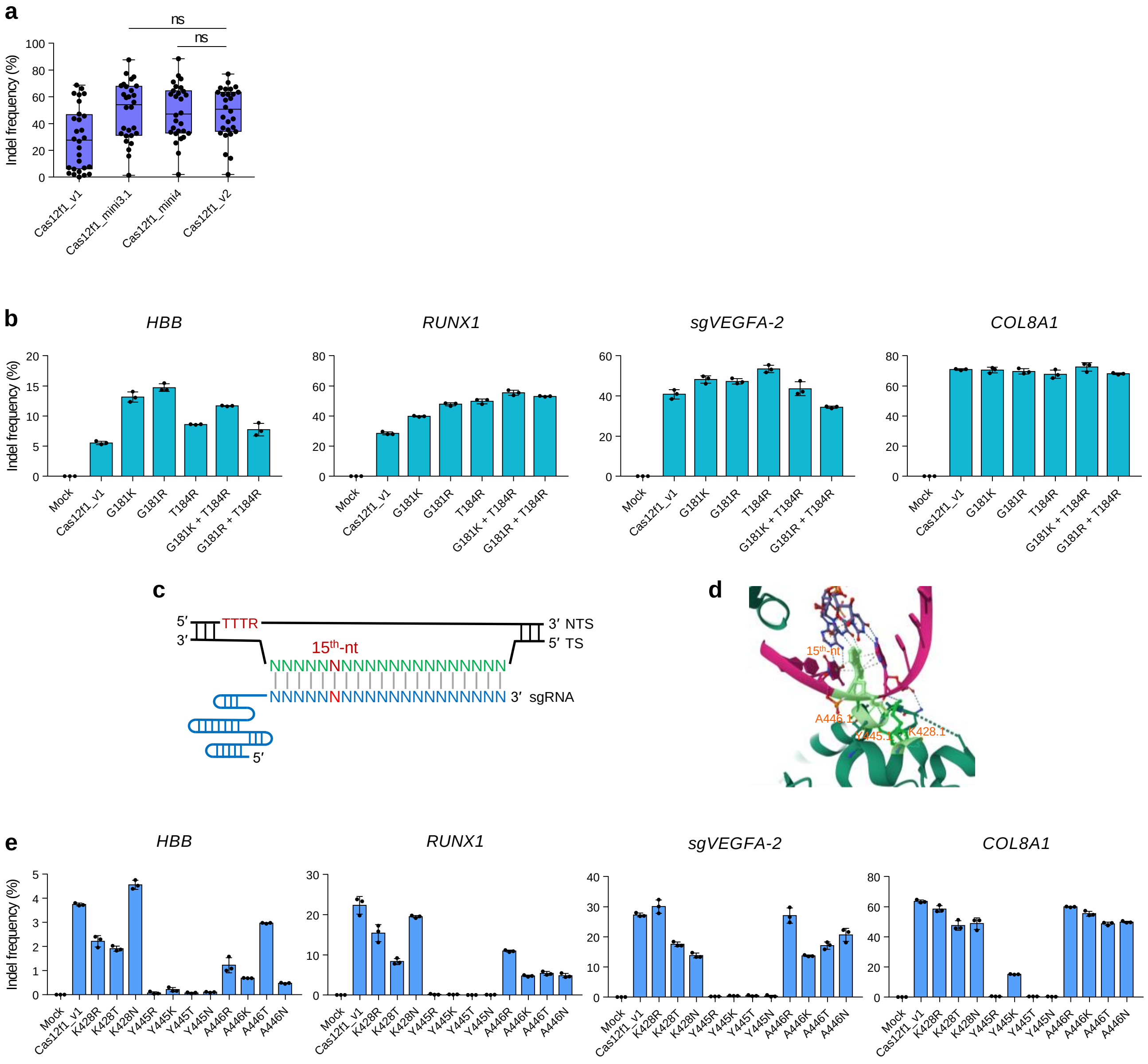

Supplementary Fig. 3 Comparison of gene editing efficiency of Cas12f1 variants with mutated amino acids that interact with the 6th nucleotide of the sgRNA spacer or the 15th nucleotide of the target DNA strand.

a Indel efficiency among Cas12f1_v1, Cas12f1_mini3.1, Cas12f1_mini4 and Cas12f1_v2 at twenty-eight gene targets. **b** Evaluation of gene editing efficiency of Cas12f1 variants with G181 and T184 mutation combinations at four targets. **c** Position of the 15th nucleotide in the target strand. TS, target strand; NTS, non-target strand; magenta letters, PAM sequence; light green letters, target sequence; light blue letters, sgRNA spacer sequence. **d** Location of K428, Y445, and A446 in Cas12f1.1. K428, Y445, A446, and the 15th nucleotide are highlighted in light green (PDB: 7C7L). **e** Assessment of indel efficiency among eleven Cas12f1 mutants that include amino acid replacement at K428, Y445, or A446. In the box-and-whisker plot, boxes represent the interquartile ranges with the median as a line, and whiskers extend from the minimum to the maximum values. Data in bar graphs represent the mean ± s.d. of three independent biological replicates. *P*-values were obtained using the two-tailed Student's t-test. ns, not significant.

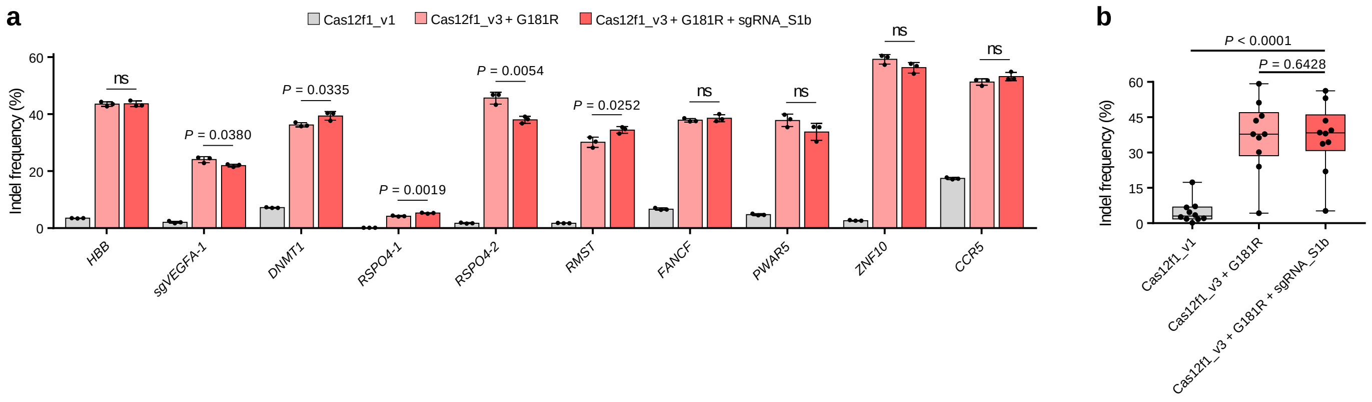

Supplementary Fig. 4 Indel frequencies of Cas12f1_v3 + G181R and Cas12f1_v3 + G181R + sgRNA_S1b variants.

a,**b** Comparison of indel efficiencies between Cas12f1_v3 + G181R and Cas12f1_v3 + G181R + sgRNA_S1b at ten gene targets. Data in the bar graphs represent the mean ± s.d. of three independent biological replicates. In the box-and-whisker plot, boxes represent the interquartile ranges with the median as a line, and whiskers extend from the minimum to the maximum values. *P*-values were obtained using the two-tailed Student’s t-test. ns, not significant.

Engineered Cas12f	Mutations introduced
enAsCas12f	D196K, N199K, G276R, N328G, D364R
AsCas12f-YHAM	F48Y, S188H, V232A, E316M
AsCas12f1-HKRA	I123H, D195K, D208R, V232A
enOsCas12f1	D52R, T132R
enRhCas12f1	L270R

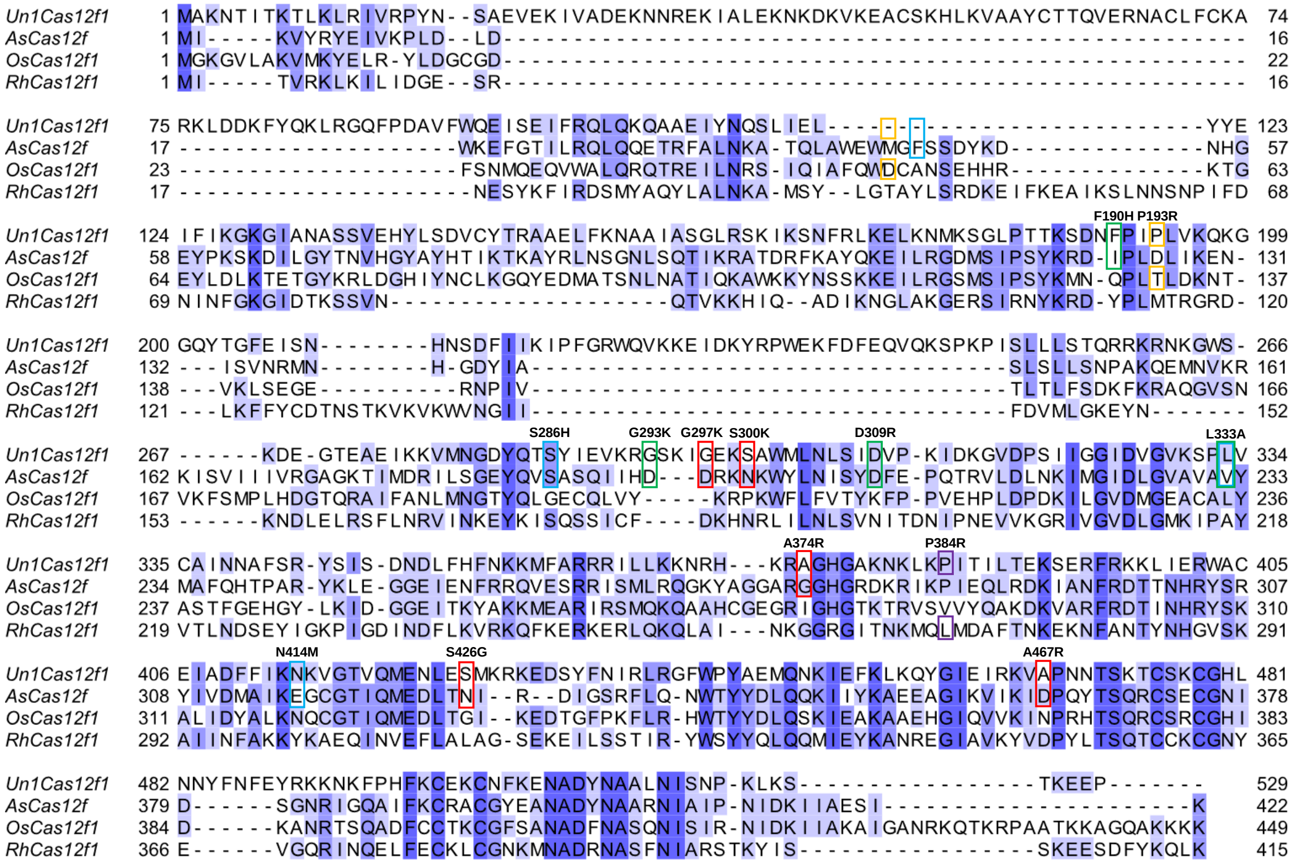

Supplementary Fig. 5 Protein sequence alignment of Un1Cas12f1, AsCas12f, OsCas12f1, and RhCas12f1.

The multiple sequence alignment was performed using the T-Coffee and visualized using Jalview[24,25]. The table above shows the engineered Cas12f variants and their mutations introduced to improve the gene editing efficiency of AsCas12f, OsCas12f1, and RhCas121. Each mutation is highlighted in the alignment using colors designated for the respective Cas12f variants: red for enAsCas12f, light blue for AsCas12f-YHAM, green for AsCas12f-HKRA, yellow for enOsCas12f1, and purple for enRhCas12f1. The amino acid positions in Un1Cas12f1 corresponding to the mutations introduced in each enhanced Cas12f variants are indicated above the Un1Cas12f1 sequence.

a

Candidate mutations for Cas12f1_v7		
enOsCas12f1	D52R	T132R
Cas12f1	-	P193R
enRhCas12f1	L270R	
Cas12f1	P384R	

b

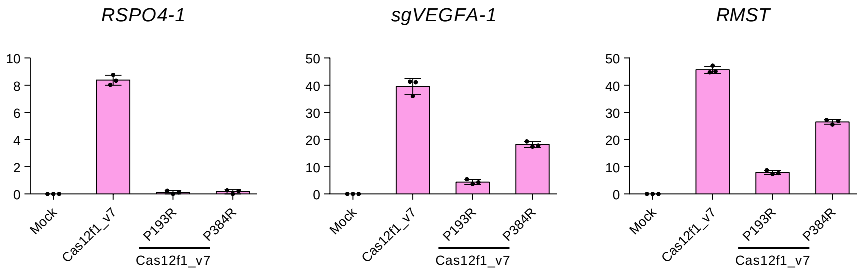

Supplementary Fig. 6 Indel efficiency of Cas12f1_v7 variants with mutations introduced to enOsCas12f1 and enRhCas12f1.

a Mutations introduced to enhance the gene editing efficiency of OsCas12f1 and RhCas12f1 and the corresponding Cas12f1 amino acids.
b Comparison of indel efficiencies of Cas12f1_v7 variants with each mutations in a.

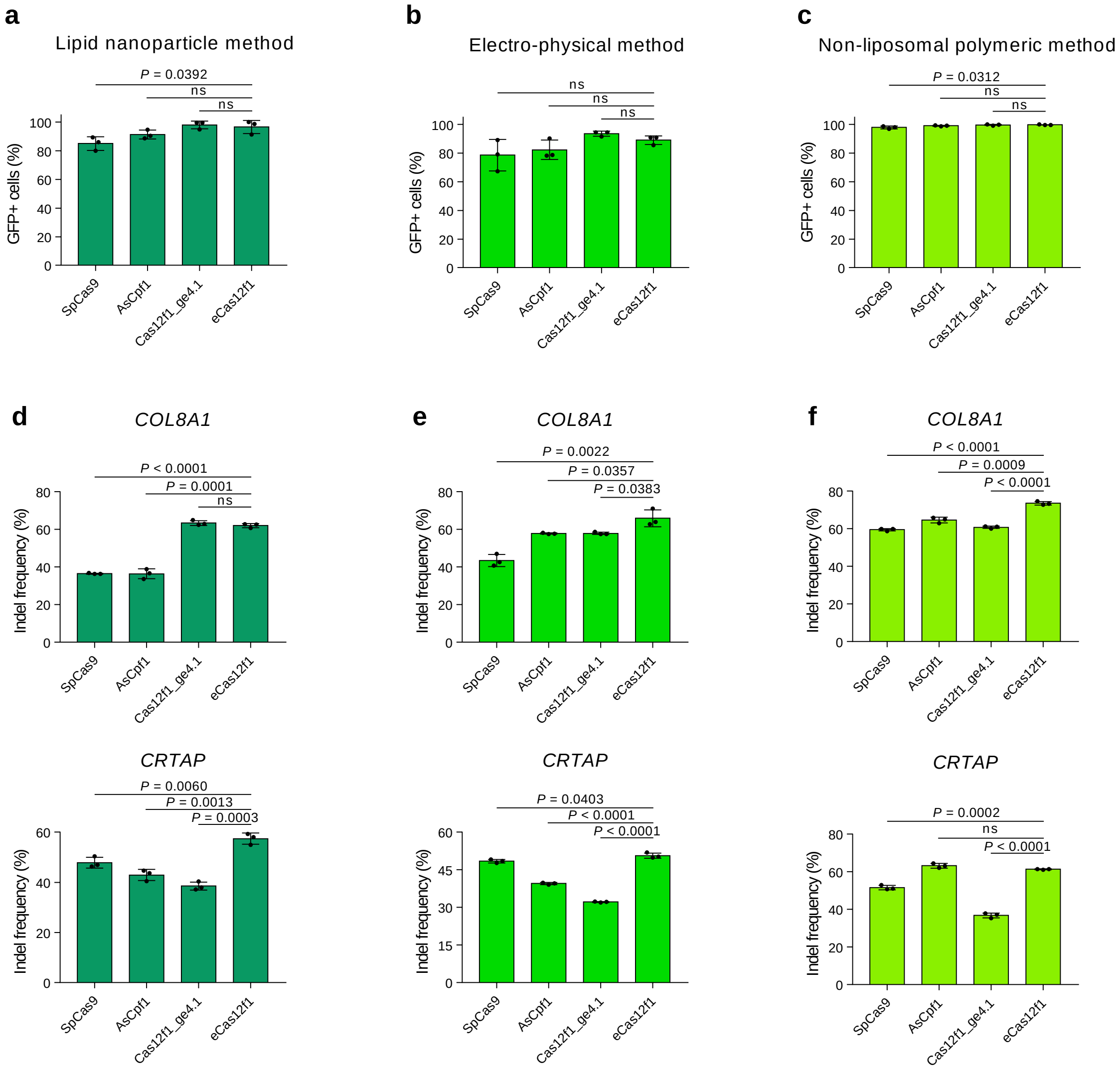

Supplementary Fig. 7 Comparison of intracellular delivery and gene editing efficiency of SpCas9, AsCpf1, Cas12f1_ge4.1, and eCas12f1 using three different DNA delivery methods.

a-**c** Transfection efficiency of lipid nanoparticle method (Lipofectamine™ 3000 Transfection Reagent) (a), electro-physical method (Neon™ Transfection System) (b), and non-liposomal polymeric method (TransIT-X2 Dynamic Delivery System) (c) in HEK293T cells. Plasmids encoding each CRISPR system and EGFP reporter gene were used at the same copy number and transfection efficiency was calculated by counting GFP-positive HEK293T cells from 10,000 events after 72 hr post-transfection. **d**-**f** Indel frequency of each CRISPR system transfected using lipid nanoparticle method (d), electro-physical method (e), or non-liposomal polymeric method (f). Data represent the mean ± s.d. of three independent biological replicates. *P*-values were obtained using the two-tailed Student's t-test. ns, not significant.

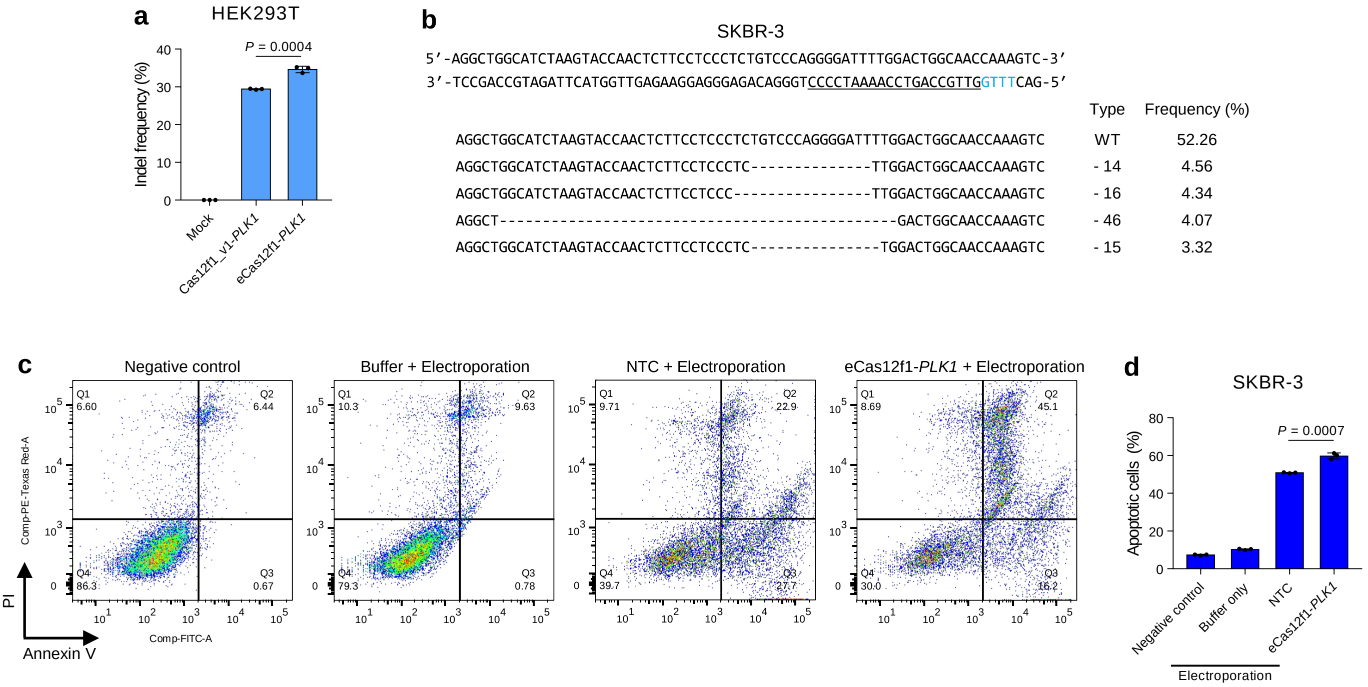

Supplementary Fig. 8 Evaluation of eCas12f1 as a potential breast cancer therapy.

a Gene editing efficiency of eCas12f1 targeting *PLK1* in HEK293T cells. The indel frequency was analyzed from transfected cell pool. **b** Genotypes of *PLK1* in SKBR-3 after treatment with eCas12f1-*PLK1*. The sequences with the top five number of reads are displayed. Underline indicates the sequence of the sgRNA spacer; light blue letters indicate the PAM sequence. **c** Flow cytometry analysis of SKBR-3 cells following treatment with eCas12f1-*PLK1* and dual staining with annexin V and PI for cell apoptosis assay. **d** The SKBR-3 cells treated with eCas12f1-*PLK1* showed 8.90% higher rate of apoptotic cells compared to those treated with non-targeting eCas12f1. NTC, non-targeting control. Data in the bar graphs represent the mean ± s.d. of three independent biological replicates. *P*-values were obtained using the two-tailed Student’s t-test.

a

b

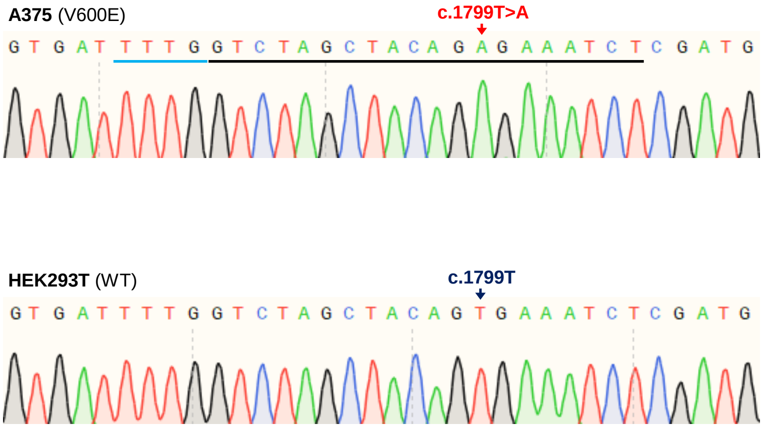

Supplementary Fig. 9 Genotypes of *BRAF* gene in A375 and HEK293T cells.

a Genotypes of A375 cells carrying c.1799T>A mutation in exon15 of *BRAF* gene (red arrow), resulting in a missense mutation (V600E). The PAM and sgRNA spacer sequence targeting *BRAF* mutation were underlined with light blue and black, respectively. **b** Genotypes of HEK293T cells harboring wild type *BRAF* gene. WT, wild type.

a

	A1	A2	A3	A4	A5	A6	A7	A8	A9	A10	A11	A12	A13	A14	A15	A16	A17	A18	A19	A20
NLRC4		1.7				28		8		22		74	2.3					4.4		4.1
sgVEGFA-2			98.1					38		33	2.5	7.2		70						
CXCR4	0.0		90.5	50.4		43.5							70.3		6.2	0.9				
CDKL5		20.0						24.1			64.6		58.0		3.6				19.0	0.5
HEK4		5.1	92	93		16				15		64.2			4.3					2.0
PWAR5	0.0	0.8		95.4	69.3	14.6			47.1				2.8			22.7	12.4			0.4
RSPO4-1	0.2				76.8		55.7		24.8			24.5								
RSPO4-2	0.2	13.2			88.3	8.6	4.0											12.3		

A-to-G conversion frequency over edited reads (%)

b

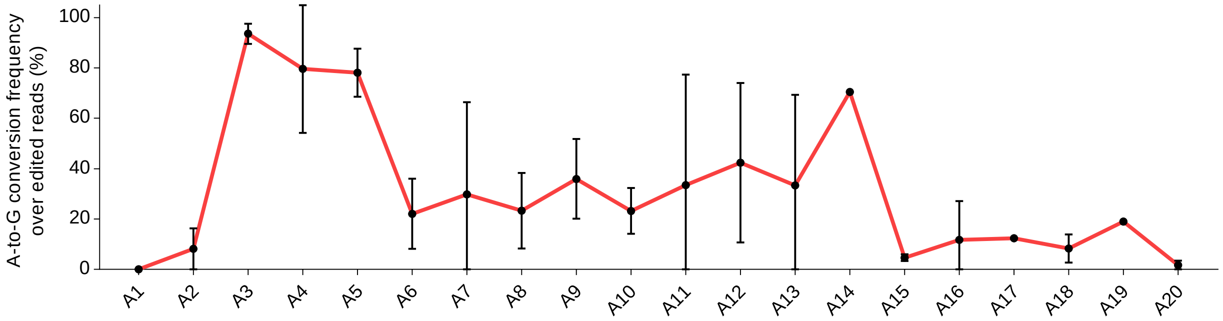

Supplementary Fig. 10 Base editing window of eCas12f1-ABE.

a Base editing frequency of eCas12f1-ABE at adenines for eight target sites. The data shown are the percentage of the number of reads with A-to-G conversion at a specified position over the total number of edited reads. **b** Base editing activity window for eCas12f1-ABE across eight target sites. Dots represent an average of the percentages shown in **a** at the indicated base positions and error bars indicate ± s.d. of the percentages across the eight targets.

a

	C1	C2	C3	C4	C5	C6	C7	C8	C9	C10	C11	C12	C13	C14	C15	C16	C17	C18	C19	C20
CLIC4	1.5	10.4	83.5				33.4			6.4	52.2		42.9	21.2	0.0	21.0			0.0	5.2
COL8A1					73.6				32.7		60.7					2.9	2.1			
RNF2	4.5		88.6			20.3		10.3						0.0	0.1	1.3	3.7			
sgVEGFA-3	0.1		98.4	86.5					4.6	3.4	5.3	3.6	3.9						12.2	
DNMT1		66.5						19.9				0.0	5.4	31.3	0.0					4.7
CDKL5	20.5				65.6		47.4		0.0	0.0		0.0					53.6			
CARS1	2.4			72.9			1.9	7.4		55.1		3.5	6.3						1.8	

C-to-T conversion frequency over edited reads (%)

b

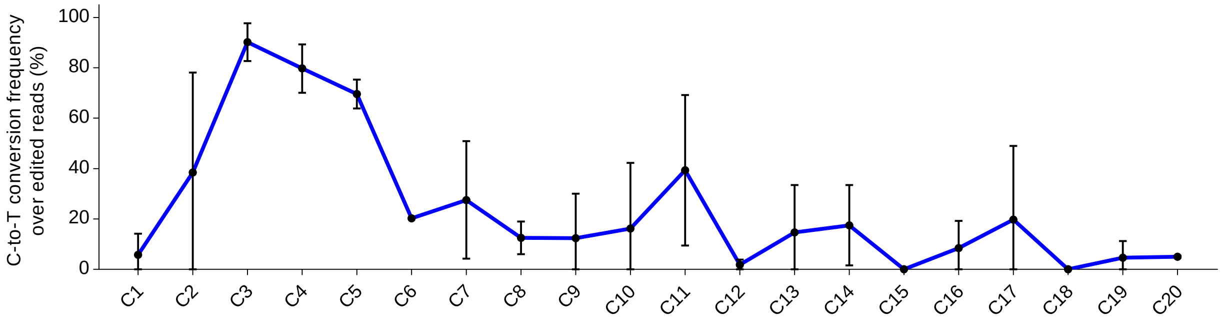

Supplementary Fig. 11 Base editing window of eCas12f1-CBE.

a Base editing frequency of eCas12f1-CBE at cytosines for seven target sites. The data shown are the percentage of the number of reads with C-to-T conversion at a specified position over the total number of edited reads. **b** Base editing activity window for eCas12f1-CBE across seven target sites. Dots represent an average of the percentages shown in **a** at the indicated base positions and error bars indicate ± s.d. of the percentages across the seven targets.

Supplementary Table 1. sgRNA spacer sequences for evaluating Cas12f1 variants.

NO.	Target gene	PAM sequence (5′-3′)	Spacer sequence (5′-3′)
1	*NLRC4*	TTTA	GAGGGAGACACAAGTTGATA
2	*CLIC4*	TTTA	CCCTGGCTACCTCCCCTACC
3	*HBB*	TTTG	CCAAAGTGATGGGCCAGCAC
4	*PLK1*	TTTG	GTTGCCAGTCCAAAATCCCC
5	*COL8A1*	TTTA	GATTCATTCTCAGTGCCATG
6	*HEK3*	TTTG	GCTGGGCTGGAAGCCAGCAC
7	*RNF2*	TTTA	CACGTCTCATATGCCCCTTG
8	*VEGFA*	TTTG	CTCCCGGCCGCTGGTCCCGG
9	*TERT*	TTTG	TGCTGGTGGCTCCCAGCTGC
10	*sgVEGFA-1*	TTTG	GGACTGGAGTTGCTTCATGT
11	*sgVEGFA-2*	TTTG	GGAGGTCAGAAATAGGGGGT
12	*sgVEGFA-3*	TTTG	CTCCTGGACCCCCTATTTCT
13	*LRP5*	TTTA	CATTGTGGGGCCTTGTCTGG
14	*KLHL29*	TTTA	GAGAGACCGCTCAGGCTGGA
15	*CRTAP*	TTTG	CAGGGCTTGGGATTTGTCTT
16	*EMX1*	TTTA	CCATAGAGTCCTTGGTGGCC
17	*FANCF*	TTTG	GGTCTCAGCGCAGGCCTCAG
18	*RUNX1*	TTTA	ATGACCTCAGGTTTGTCGGT
19	*CCR5*	TTTG	TGGGCAACATGCTGGTCATC
20	*CXCR4*	TTTG	AGAACACTGTGCACAAGTGG
21	*CDLK5*	TTTG	CAGTCTCACCACAGATCTAA
22	*HEK4*	TTTA	CAAAGATTGACACCATTGCA
23	*DNMT1*	TTTA	TCGTATACTGACCCCTTTGC
24	*PWAR5*	TTTA	AACAAATCACTGACTAACCA
25	*RMST*	TTTA	ATAATGCCTTTTAGGTGATA
26	*ZNF10*	TTTA	ATTCCCACAATAACCCTATG
27	*RSPO4-1*	TTTA	ACTCATACATCACCTCCTCC
28	*RSPO4-2*	TTTA	AAGGAAAGGCTTCCTGGAGG

Supplementary Table 2. Target sequences used for comparison between SpCas9, AsCpf1, CasMINI-V3.1, Cas12f1_ge4.1, and eCas12f1.

NO.	Target gene	TTTR (5′-3′)	N_{20} DNA (5′-3′)	NGG (5′-3′)
1	*CLIC4*	TTTA	CCCTGGCTACCTCCCCTACC	CGG
2	*COL8A1*	TTTA	GATTCATTCTCAGTGCCATG	GGG
3	*CRTAP*	TTTG	CAGGGCTTGGGATTTGTCTT	GGG
4	*KLHL29*	TTTA	GAGAGACCGCTCAGGCTGGA	GGG
5	*NLRC4*	TTTA	GAGGGAGACACAAGTTGATA	GGG
6	*PWAR5*	TTTA	AACAAATCACTGACTAACCA	AGG
7	*RMST*	TTTA	ATAATGCCTTTTAGGTGATA	AGG
8	*RSPO4-1*	TTTA	ACTCATACATCACCTCCTCC	AGG
9	*RSPO4-2*	TTTA	AAGGAAAGGCTTCCTGGAGG	AGG
10	*TYW1B*	TTTA	GATCCGATGCAATTTTGGGA	AGG
11	*ZNF10*	TTTA	ATTCCCACAATAACCCTATG	AGG
12	*CARS1*	TTTA	CAACAGCCTCACCAGGAACA	AGG
13	*NEK10-1*	TTTA	AGACAAGCTGTCTTCCTTCA	GGG
14	*NEK10-2*	TTTA	ATCTGAAGATCATTGAAACA	GGG
15	*BCL2L13*	TTTA	ATTTCCAAGTCAACCTTATG	AGG
16	*AHRR*	TTTA	CCTTAATAAAGTATAACTTC	AGG
17	*APEX1*	TTTA	AAGAAGGAATGGTAGTTGAG	GGG
18	*GAB4*	TTTA	CCTGGTGGCTGAGACCAGGG	AGG
19	*POLRMT-1*	TTTA	GAAACTGCCCCAAAACCGGC	CGG
20	*POLRMT-2*	TTTA	AGGACTATGTGTGGCCAGTG	AGG
21	*UNCX*	TTTA	CCTGAACTCGGGACTCGACC	AGG
22	*RPH3AL-1*	TTTA	ATTTTCAAAACAGCCCTATG	GGG
23	*RPH3AL-2*	TTTA	CACAAGGGATCTGAGACTTG	AGG
24	*ZMYM2*	TTTA	GTAGGCTGCTGTTGGACAGA	CGG
25	*WNK1*	TTTA	GAACCCAGTGAAAAATACCA	GGG
26	*NLGN1*	TTTA	GTCTAATAGAAATATAGTAC	AGG
27	*MLH1*	TTTA	GATCAATTTACATCAAACTA	GGG

Supplementary Table 3. Target sequences used for regulation of gene expression.

Application	Target gene	PAM sequence (5′-3′)	Spacer sequence (5′-3′)
eCas12f1a	*IL1RN*	TTTG	GTTTCTGCTAGCCTGAGTCA
	IFNγ	TTTA	CCAGGGCGAAGTGGGGAGGT
	HBG	TTTG	CATTGAGATAGTGTGGGGAA
eCas12f1i	*HER2*	TTTA	CTAGAGGATGTGGTGGGAAA
	CHK1	TTTA	GAAACCTTCCCTACTCTACC
	HBG	TTTG	CATTGAGATAGTGTGGGGAA

Supplementary Table 4. Primer sequences for real-time qPCR.

Target gene	Forward primer (5′-3′)	Reverse primer (5′-3′)
IL1RN	GGAATCCATGGAGGGAAGAT	TGTTCTCGCTCAGGTCAGTG
IFNγ	GAGTGTGGAGACCATCAAGGA	TGTATTGCTTTGCGTTGGAC
HBG	GCTGAGTGAACTGCACTGTGA	GAATTCTTTGCCGAAATGGA
HER2	GGGAAACCTGGAACTCACCT	GACCTGCCTCACTTGGTTGT
CHK1	ATATGAAGCGTGCCGTAGACT	TGCCTATGTCTGGCTCTATTCTG
GAPDH	CAATGACCCCTTCATTGACC	TTGATTTTGGAGGGATCTCG

Supplementary Table 5. DNA sequences of eCas12f1.

eCas12f1 (5′-3′)
Purple : Bipartite NLS<br>Blue : eCas12f1<br><br>AAACGGACAGCCGACGGAAGCGAGTTCGAGTCACCAAAGAAGAAGCGGAAAGTCGCCAAGAACACAATCACCAAGACACTGAAGCTGAGAATC<br>GTGCGGCCTTACAACAGCGCCGAGGTGGAAAAAATCGTGGCCGATGAGAAGAACAACAGAGAGAAAATCGCTCTGGAAAAAAATAAGGACAAG<br>GTTAAAGAAGCCTGCTCCAAGCACCTGAAAGTGGCTGCCTACTGCACCACCCAGGTCGAGAGAAACGCCTGTCTGTTCTGCAAGGCCAGAAAGC<br>TGGACGACAAGTTCTACCAAAAGCTGAGAGGCCAGTTCCCCGACGCCGTGTTCTGGCAGGAGATTAGCGAGATTTTCAGACAGCTGCAGAAGCA<br>GGCCGCCGAGATCTACAACCAGAGCCTGATCGAGCTGTACTACGAGATCTTTATCAAAGGCAAAGGAATTGCCAACGCTTCTAGCGTGGAACAC<br>TACCTGAGCAGAGTGTGCTACCGGAGAGCCGCCGAGCTGTTCAAGAATGCCGCTATCGCCAGCGGACTGCGGAGCAAGATCAAGTCTAACTTC<br>CGGCTGAAGGAACTCAAGAATATGAAATCCCGGCTGCCAACAACAAAGAGCGACAACTTTCCAATCCCCCTGGTGAAGCAGAAAGGAGGCCAGT<br>ATACCGGCTTTGAGATCAGCAACCACAACTCTGATTTCATCATCAAGATCCCATTCGGCAGATGGCAAGTGAAGAAGGAAATCGACAAGTACAGA<br>CCTTGGGAGAAGTTCGACTTCGAGCAGGTGCAGAAGAGTCCTAAGCCTATCAGCCTGCTGCTGTCTACCCAGAGACGGAAACGGAACAAAGGCT<br>GGTCCAAGGACGAGGGCACCGAAGCCGAAATCAAGAAGGTGATGAACGGCGACTACCAGACCAGCTACATCGAAGTGAAGAGGGGCAGCAAG<br>ATCGGCGAGAAGTCTGCCTGGATGCTGAACCTGAGCATCGATGTTCCTAAGATCGACAAAGGAGTGGACCCCAGCATCATCGGCGGAATTGATG<br>TGGGCGTGAGAAGCCCTCTGGTCTGTGCCATCAACAATGCTTTTAGCAGATACAGCATCTCCGATAACGACCTGTTTCACTTTAACAAGAAGATG<br>TTCGCCAGGCGGAGAATCCTGCTTAAAAAGAACCGCCACAAGAGAGCCGGCCACGGCGCCAAGAACAAGCTCAAACCTATCACCATCCTGACCG<br>AGAAGAGCGAGCGGTTCCGGAAAAAGCTGATCGAGAGATGGGCCTGTGAAATCGCCGACTTCTTCATCAAAAATAAGGTGGGCACAGTGCAGAT<br>GGAAAACCTGGAAGGCATGAAGAGAAAGGAAGATAGCTATTTCAACATCCGGCTGAGAGGCTTCTGGCCTTACGCCGAGATGCAGAACAAGATC<br>GAGTTCAAGCTGAAGCAATACGGCATCGAGATCAGAAAGGTGGCTCCTAACAACACCAGCAAAACCTGTAGCAAGTGCGGCCATCTGAACAACT<br>ACTTCAACTTCGAGTACAGAAAGAAGAACAAGTTCCCCCACTTCAAGTGCGAGAAGTGCAACTTCAAGGAAAACGCCGATTATAATGCTGCGCTG<br>AATATCTCTAATCCTAAGCTGAAATCTACAAAGGAGGAACCC
