# Supplementary material for: Robust genome editing activity and the applications of enhanced miniature CRISPR-Cas12f1
Source: Nat Commun. 2025 Jan 15;16:677. doi: 10.1038/s41467-025-56048-w (PMC11733285; doi:10.1038/s41467-025-56048-w)
Supplement: Supplementary file 2 — Description of Additional Supplementary Files [file 41467_2025_56048_MOESM2_ESM.pdf]

Description of Additional Supplementary Files

File Name: Supplementary Data 1
Description: Primer sequences for analysis of gene editing and base editing efficiency.
