# Supplementary material for: Robust genome editing activity and the applications of enhanced miniature CRISPR-Cas12f1
Source: Nat Commun. 2025 Jan 15;16:677. doi: 10.1038/s41467-025-56048-w (PMC11733285; doi:10.1038/s41467-025-56048-w)
Supplement: Supplementary file 4 — Reporting Summary [file 41467_2025_56048_MOESM4_ESM.pdf]

nature portfolio

Corresponding author(s): DAPR (NCOMMS-24-23143A)

Last updated by author(s): Dec 18, 2024

Reporting Summary

Nature Portfolio wishes to improve the reproducibility of the work that we publish. This form provides structure for consistency and transparency in reporting. For further information on Nature Portfolio policies, see our Editorial Policies and the Editorial Policy Checklist.

Statistics

For all statistical analyses, confirm that the following items are present in the figure legend, table legend, main text, or Methods section.

n/a	Confirmed	
☐	☒	The exact sample size (*n*) for each experimental group/condition, given as a discrete number and unit of measurement
☐	☒	A statement on whether measurements were taken from distinct samples or whether the same sample was measured repeatedly
☐	☒	The statistical test(s) used AND whether they are one- or two-sided<br>*Only common tests should be described solely by name; describe more complex techniques in the Methods section.*
☒	☐	A description of all covariates tested
☒	☐	A description of any assumptions or corrections, such as tests of normality and adjustment for multiple comparisons
☐	☒	A full description of the statistical parameters including central tendency (e.g. means) or other basic estimates (e.g. regression coefficient) AND variation (e.g. standard deviation) or associated estimates of uncertainty (e.g. confidence intervals)
☐	☒	For null hypothesis testing, the test statistic (e.g. *F*, *t*, *r*) with confidence intervals, effect sizes, degrees of freedom and *P* value noted<br>*Give P values as exact values whenever suitable.*
☒	☐	For Bayesian analysis, information on the choice of priors and Markov chain Monte Carlo settings
☒	☐	For hierarchical and complex designs, identification of the appropriate level for tests and full reporting of outcomes
☒	☐	Estimates of effect sizes (e.g. Cohen's *d*, Pearson's *r*), indicating how they were calculated

Our web collection on statistics for biologists contains articles on many of the points above.

Software and code

Policy information about availability of computer code

Data collection	Deep sequencing and GUIDE-seq data were collected using MiSeq system (Illumina). BD FACSCanto™ II and BD FACSAria™ III(BD Biosciences) was used to collect flow cytometry data. CFX Connect Real-Time PCR Detection System (BioRad) used to collect quantitative real-time PCR data. ECLIPSE Ti2-U (Nikon) was used to collect cell images.
Data analysis	EUN (https://daeunyoon.com) was used for NGS analysis. Microsoft Excel (v2410) and Graphpad prism 7 were used for analysis of indel efficiency. GUIDE-seq analysis package (v1.0.2, https://github.com/aryeelab/guideseq) was used for off-target analysis. Flowjo (v10.10.0) was used for transfection efficiency and apoptotic cell analysis. Bio-Rad CFX Manager 3.1 software was used for analysis of mRNA expression level, RNAeval web server (http://rna.tbi.univie.ac.at/cgi-bin/RNAWebSuite/RNAeval.cgi) was used for free energy calculation of sgRNAs, WebLogo (v2.8.2, https://weblogo.berkeley.edu/logo.cgi) was used to create sequence logo. ImageJ 1.52 was used for analysis of colony formation assay, Jalview (v2.11.4.1) was used for protein sequence alignment. NIS-Elements BR (v5.11.00) was used to analyze GFP+ HEK293T cells.

For manuscripts utilizing custom algorithms or software that are central to the research but not yet described in published literature, software must be made available to editors and reviewers. We strongly encourage code deposition in a community repository (e.g. GitHub). See the Nature Portfolio guidelines for submitting code & software for further information.

Data

Policy information about availability of data

All manuscripts must include a data availability statement. This statement should provide the following information, where applicable:

- Accession codes, unique identifiers, or web links for publicly available datasets
- A description of any restrictions on data availability
- For clinical datasets or third party data, please ensure that the statement adheres to our policy

The NGS data and GUIDE-seq generated in this study have been deposited in the NCBI Sequence Read Archive (SRA) under accession number PRJNA1104538 [https://www.ncbi.nlm.nih.gov/sra/?term=PRJNA1104538]. All data supporting the findings of this study are provided within the paper and Supplementary Information. Source data are provided with this paper.

Research involving human participants, their data, or biological material

Policy information about studies with human participants or human data. See also policy information about sex, gender (identity/presentation), and sexual orientation and race, ethnicity and racism.

Reporting on sex and gender	N/A
Reporting on race, ethnicity, or other socially relevant groupings	N/A
Population characteristics	N/A
Recruitment	N/A
Ethics oversight	N/A

Note that full information on the approval of the study protocol must also be provided in the manuscript.

Field-specific reporting

Please select the one below that is the best fit for your research. If you are not sure, read the appropriate sections before making your selection.

☒ Life sciences ☐ Behavioural & social sciences ☐ Ecological, evolutionary & environmental sciences

For a reference copy of the document with all sections, see nature.com/documents/nr-reporting-summary-flat.pdf

Life sciences study design

All studies must disclose on these points even when the disclosure is negative.

Sample size	No statistical method was used to predetermine sample size. Sample sizes were determined based on our previous experimental results in related studies of genome editing (https://doi.org/10.1186/s13059-021-02389-w).
Data exclusions	No data was excluded.
Replication	All experiments were performed in three biologically independent replications and all attempts at replication were successful as provided within the paper, Supplementary Information, and Source Data.
Randomization	The experiments were not randomized because no human participants or animal subjects were involved in this study.
Blinding	The investigators were not blinded to group allocation during data collection and analysis because no group allocation was involved in this study.

Reporting for specific materials, systems and methods

We require information from authors about some types of materials, experimental systems and methods used in many studies. Here, indicate whether each material, system or method listed is relevant to your study. If you are not sure if a list item applies to your research, read the appropriate section before selecting a response.

Materials & experimental systems

n/a	Involved in the study
☒	☐ Antibodies
☐	☒ Eukaryotic cell lines
☒	☐ Palaeontology and archaeology
☒	☐ Animals and other organisms
☒	☐ Clinical data
☒	☐ Dual use research of concern
☒	☐ Plants

Methods

n/a	Involved in the study
☒	☐ ChIP-seq
☐	☒ Flow cytometry
☒	☐ MRI-based neuroimaging

Eukaryotic cell lines

Policy information about cell lines and Sex and Gender in Research

Cell line source(s)	HEK293T (ATCC, CRL-3216), SKBR-3 (ATCC, HTB-30), A375 (ATCC, CRL-1619)
Authentication	Each cell line was authenticated by morphological observation and confirmation of genotypes using NGS.
Mycoplasma contamination	All cell lines tested for mycoplasma contamination and found to be negative.
Commonly misidentified lines (See ICLAC register)	No commonly misidentified cell lines used.

Plants

Seed stocks	N/A
Novel plant genotypes	N/A
Authentication	N/A

Flow Cytometry

Plots

Confirm that:

☒ The axis labels state the marker and fluorochrome used (e.g. CD4-FITC).

☒ The axis scales are clearly visible. Include numbers along axes only for bottom left plot of group (a 'group' is an analysis of identical markers).

☒ All plots are contour plots with outliers or pseudocolor plots.

☒ A numerical value for number of cells or percentage (with statistics) is provided.

Methodology

Sample preparation	Cells were dissociated into single cells using 0.25% trypsin-EDTA and washed with DPBS. Then the cells were resuspended in DPBS.
Instrument	BD FACSCanto™ II and BD FACSAria™ III (BD Biosciences).
Software	Flowjo (v10.10.0)
Cell population abundance	10,000 events were recorded per sample.
Gating strategy	Relevant gating strategies were provided in Supplementary Figure 1a. GFP+ cells were counted after gating live and singlet cells.

☒ Tick this box to confirm that a figure exemplifying the gating strategy is provided in the Supplementary Information.
